# RTP801/REDD1 contributes to neuroinflammation severity and memory impairments in Alzheimer’s disease

**DOI:** 10.1038/s41419-021-03899-y

**Published:** 2021-06-15

**Authors:** Leticia Pérez-Sisqués, Anna Sancho-Balsells, Júlia Solana-Balaguer, Genís Campoy-Campos, Marcel Vives-Isern, Ferran Soler-Palazón, Marta Anglada-Huguet, Miguel-Ángel López-Toledano, Eva-Maria Mandelkow, Jordi Alberch, Albert Giralt, Cristina Malagelada

**Affiliations:** 1 Departament de Biomedicina, Facultat de Medicina i Ciències de la Salut, Institut de Neurociències, Universitat de Barcelona, Barcelona, Catalonia Spain; 2 Institut d’Investigacions Biomèdiques August Pi i Sunyer (IDIBAPS), Barcelona, Catalonia Spain; 3 Centro de Investigación Biomédica en Red sobre Enfermedades Neurodegenerativas (CIBERNED), Madrid, Spain; 4 German Center for Neurodegenerative Diseases (DZNE), Bonn, Germany; 5 CAESAR Research Center, Bonn, Germany; 6 Neurelis, Inc., San Diego, CA USA; 7 Production and Validation Center of Advanced Therapies (Creatio), Faculty of Medicine and Health Science, University of Barcelona, Barcelona, Catalonia Spain

**Keywords:** Alzheimer's disease, Alzheimer's disease

## Abstract

RTP801/REDD1 is a stress-regulated protein whose upregulation is necessary and sufficient to trigger neuronal death. Its downregulation in Parkinson’s and Huntington’s disease models ameliorates the pathological phenotypes. In the context of Alzheimer’s disease (AD), the coding gene for RTP801, *DDIT4*, is responsive to Aβ and modulates its cytotoxicity in vitro. Also, RTP801 mRNA levels are increased in AD patients’ lymphocytes. However, the involvement of RTP801 in the pathophysiology of AD has not been yet tested. Here, we demonstrate that RTP801 levels are increased in postmortem hippocampal samples from AD patients. Interestingly, RTP801 protein levels correlated with both Braak and Thal stages of the disease and with GFAP expression. RTP801 levels are also upregulated in hippocampal synaptosomal fractions obtained from murine 5xFAD and rTg4510 mice models of the disease. A local RTP801 knockdown in the 5xFAD hippocampal neurons with shRNA-containing AAV particles ameliorates cognitive deficits in 7-month-old animals. Upon RTP801 silencing in the 5xFAD mice, no major changes were detected in hippocampal synaptic markers or spine density. Importantly, we found an unanticipated recovery of several gliosis hallmarks and inflammasome key proteins upon neuronal RTP801 downregulation in the 5xFAD mice. Altogether our results suggest that RTP801 could be a potential future target for theranostic studies since it could be a biomarker of neuroinflammation and neurotoxicity severity of the disease and, at the same time, a promising therapeutic target in the treatment of AD.

## Introduction

Alzheimer’s disease (AD), the most common type of dementia affecting millions of people worldwide, is characterized by progressive cognitive impairment, typically beginning with memory deterioration and followed by executive dysfunction and language, visual and practical problems along with emotional and psychiatric symptoms [1, 2]. AD pathology starts in structures such as the hippocampus and the entorhinal cortex [2, 3], being the extracellular amyloid-β (Aβ) plaques and intracellular tangles of abnormally hyperphosphorylated Tau the most representative AD hallmarks [3]. Aβ and phospho-Tau accumulation over the course of the disease impair synaptic plasticity, activate an inflammatory response, and compromise neuronal survival [4, 5].

Neuroinflammation has an active role in AD pathogenesis [6–9]. It is characterized by the activation of innate immune response due to misfolded or aggregated proteins, such as Aβ, that trigger microglial and astroglial activation and the consequent release of pro-inflammatory cytokines and chemokines. Inflammasomes, intracellular sensors of danger-associated molecular patterns (DAMPs), play an important role in triggering some of these inflammatory cascades. This response is intended to be beneficial at early stages, promoting Aβ and neuron debris clearance, but if inflammation becomes chronic, it exacerbates neurodegeneration (reviewed in [10]).

The coding product of the *DDIT4* gene is a stress-induced protein called RTP801/REDD1 [11–13]. RTP801 is a negative regulator of mTOR and Akt that is necessary and sufficient for neuron death in Parkinson’s disease (PD) [14, 15]. RTP801 is also elevated in PD [14, 16] and Huntington’s disease (HD) human brains [17], suggesting an important role for this protein in both human diseases. In this line, RTP801 downregulation in the Substantia Nigra pars compacta restored motor-learning skills in a PD mouse model subjected to chronic stress [18]. Moreover, in the R6/1 mice, an HD mouse model, RTP801 silencing prevented motor impairment correcting, in turn, the alterations in synaptic plasticity [19]. Interestingly, RTP801 modulates synaptic plasticity in models of chronic unpredictable stress leading to depression [20], a co-morbid pathology associated with AD [21].

In the context of AD, *DDIT4* is an Aβ-responsive gene that modulates Aβ cytotoxicity in vitro [22, 23]. Moreover, both RTP801 mRNA and protein levels are increased in lymphocytes derived from AD patients [24]. *DDIT4* also appears as one of the differentially expressed genes in samples from the prefrontal cortex (PFC) from AD patients [25] and is one of the few genes of the mTOR pathway that might be affected by the ApoE genotype [26].

Here, we investigated whether RTP801 is involved in AD pathogenesis using human postmortem AD samples and transgenic animal models of the disease. We found that RTP801 is elevated in the hippocampus of AD patients and its levels correlated with both Braak and Thal stages of the disease. Also, RTP801 was upregulated in hippocampal synaptosomal fractions from 5xFAD mice and in samples of the entorhinal cortex from rTg4510 mice (a mouse model of tauopathy). Hippocampal neuronal silencing of RTP801 expression in the 5xFAD mouse model prevented memory impairment and abrogated astro- and microgliosis. Altogether, our results suggest a putative role of RTP801 in the inflammatory response associated with AD and frame RTP801 as a novel target in AD.

## Results

### Hippocampal RTP801 levels are increased in Alzheimer’s disease patients and rodent models and correlate with neuropathological severity

**Fig. 1:**
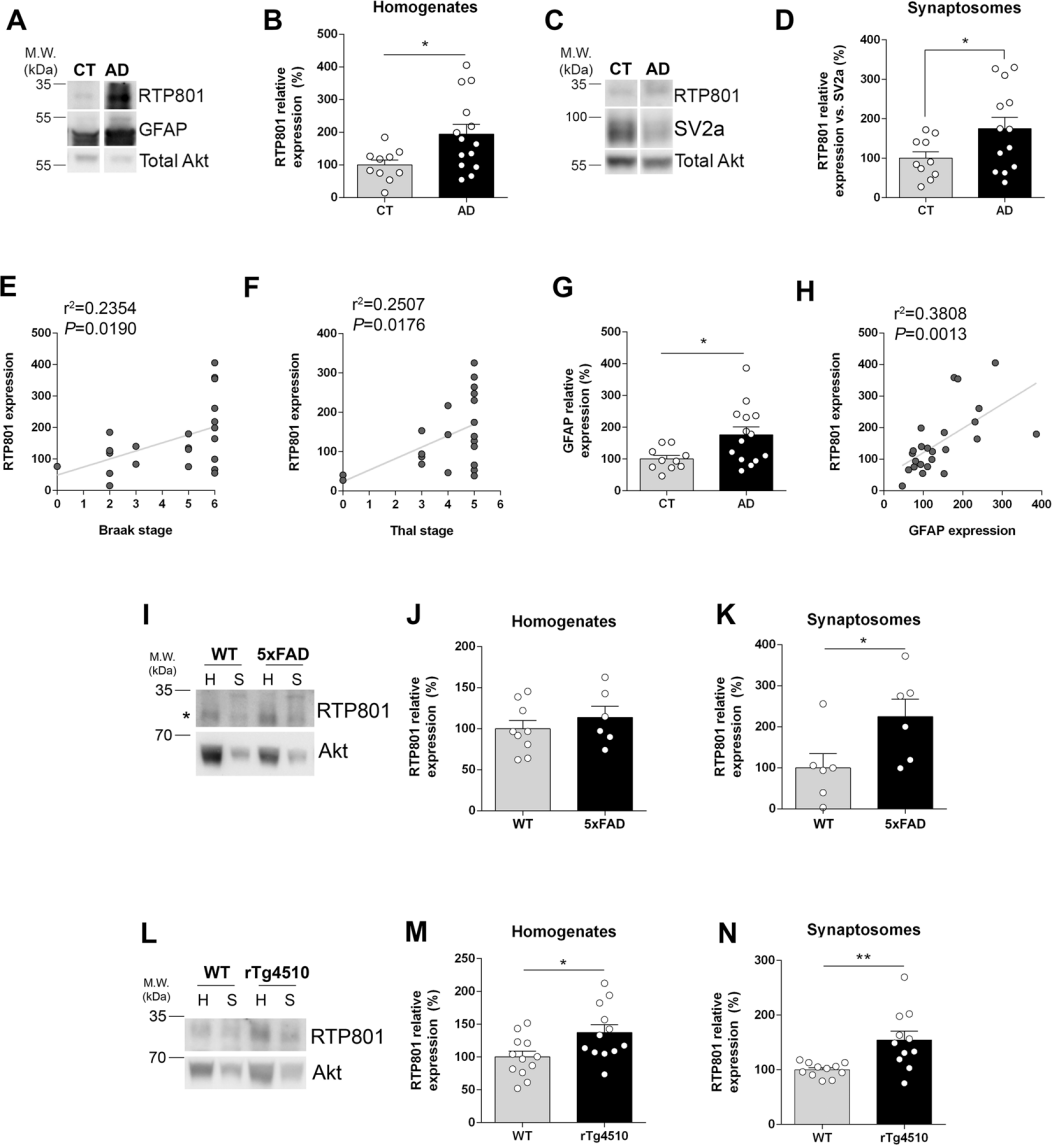
RTP801 levels in human postmortem samples with Alzheimer’s disease and 5xFAD and rTg4510 transgenic mice. **A** Immunoblotting for RTP801, GFAP, and Akt as a loading control in human postmortem hippocampal samples from controls (CT) and Alzheimer’s disease (AD) patients. **B** Densitometric quantification of RTP801 results as in (**A**) for the hippocampus (*t*_18.75_ = 2.789, *P* = 0.0118). **C** Immunoblotting for RTP801, SV2a, and Akt in the crude synaptosomal fractions from postmortem hippocampal samples from CT and AD patients. **D** Densitometric quantification of synaptic RTP801 levels normalized by SV2a levels (*t*_18.26_ = 2.254, *P* = 0.0367). **E** Pearson’s correlation analysis comparing RTP801 levels as in (**B**) with Braak stage per sample in AD patients (*P* = 0.019). **F** Pearson’s correlation analysis comparing RTP801 levels as in (**B**) with Thal stage per sample in AD patients (*P* = 0.017). **G** Densitometric quantification of GFAP results as in (**A**) for the hippocampus (*t*_17.81_ = 2.843, *P* = 0.0109). **H** Pearson’s correlation analysis comparing GFAP levels as in (**G**) with RTP801 levels as in (**B**) per sample in AD patients (*P* = 0.0013). **I** Immunoblotting for RTP801 and Akt as a loading control in total (H) and synaptosomal (S) hippocampal fractions from 5-month male and female WT and 5xFAD mice. Densitometric quantification in total (**J**) and synaptosomal (**K**) fractions of results as in (**I**) for RTP801 in 5-month-old mice (in **J**: *t*_13_ = 0.827, *P* = 0.422; in **K**: *t*_10_ = 2.254, *P* = 0.047). **l** Immunoblotting for RTP801 and Akt as a loading control in total (H) and synaptosomal (S) hippocampal fractions from 6-month male and female WT and rTg4510 mice. Densitometric quantification in total (**M**) and synaptosomal (**N**) fractions of results as in (**l**) for RTP801 in 6-month-old mice (in **M**: *t*_22_ = 2.496, *P* = 0.0206; in **N**: *t*_11.16_ = 3.284, *P* = 0.0071). In (**A**, **C**, **I**, and **L**) molecular weight markers position is indicated in kDa. In **B**, **G**, **J**, **K**, **M**, and **N** data were normalized to Akt for each sample and expressed as a percentage of the mean of WT/controls, and means and SEM are indicated. Data are analyzed with the unpaired *t* test in **B**, **D**, **G**, **J**, **K**, **M**, and **N** and with Pearson’s correlation in **E**, **F**, and **H**. In bands **B**, **D**, and **G**, *P < 0.05 compared to CT. In **K**, **M**, and **N**, *P < 0.05 and **P < 0.01 compared to WT. CT control, AD Alzheimer’s disease, WT wild-type, H total homogenates, S synaptic homogenates.

We first examined RTP801 protein levels in postmortem hippocampal human tissue (see Supplementary Table 1). In this region, total RTP801 levels were significantly increased in AD patients compared to controls (Fig. 1A, B). While RTP801 levels did not differ between CT and AD samples in synaptosomes, we detected reduced levels of the presynaptic protein SV2a in the synaptic compartment from AD patients (Supplementary Fig. 1), as previously described [27]. We observed that RTP801 protein levels were also elevated in the crude synaptosomal compartment after correcting the synaptic loss by expressing protein levels relative to the expression of the presynaptic protein SV2a (Fig. 1C, D). We next performed correlation studies in AD samples comparing RTP801 levels in the whole lysate with the Braak (Fig. 1E) and Thal (Fig. 1F) stages, respectively, for each case and we observed a significant positive correlation in both, suggesting that the neuropathological severity correlates with RTP801 levels. In addition, GFAP expression (a marker of astrogliosis [28]) was increased in AD patients (Fig. 1G) and positively correlated with RTP801 levels (Fig. 1H), reinforcing the idea of RTP801 levels as a marker of neuropathological severity.

We next analyzed the hippocampus of 5-month-old 5xFAD mice, an age at which they have an altered phenotype [29–32] comparable to the human samples. Total RTP801 levels were similar in wild-type (WT) and 5xFAD mice (Fig. 1I, J), whereas RTP801 levels in the synaptosomal fraction were higher in 5xFAD than in WT mice (Fig. 1I–K). This result indicated a functional alteration of this protein depending on its subcellular localization, specifically in the synapse in an amyloid mouse model. Next, since RTP801 levels positively correlated with Braak stages in AD human samples (Fig. 1E) we sought potential similar changes in tauopathy rodent models. We took advantage of the rTg4510 model at 6 months of age, when the phenotype of this mouse model is evident [33]. Interestingly, total RTP801 levels were significantly increased in rTg4510 mice compared with WT mice (Fig. 1L, M), and similarly, RTP801 levels in the synaptosomal fraction were also higher in rTg4510 than in WT mice (Fig. 1L–N). Altogether, our results show that RTP801 levels were increased in AD patients, 5xFAD mice, and Tg4510 mice and that such increased levels positively correlated with the severity of neurofilament tangles distribution (Braak stages), progressive deposition of amyloid-β (Thal stages), and astrogliosis.

### Genetic normalization of hippocampal RTP801 levels in 5xFAD mice induces cognitive improvements related to associative and declarative memories

We observed that RTP801 levels were widely upregulated in human hippocampal postmortem samples from AD patients, in a mouse model of tauopathy (Tg4150 mice), and the synaptosomes of a mouse model of Amyloid-β deposition (5xFAD mice). Thus, we next hypothesized that the normalization of RTP801 levels in the dorsal hippocampus of the 5xFAD mice could improve their memory deficits. To test this hypothesis, 6-month-old WT and 5xFAD mice received, in the dorsal hippocampus, a bilateral stereotactic injection of AAV expressing scramble shRNA (shCt) or shRNA against RTP801 (shRTP801) generating four groups of mice namely: WT shCt, WT shRTP801, 5xFAD shCt, and 5xFAD shRTP801.

**Fig. 2:**
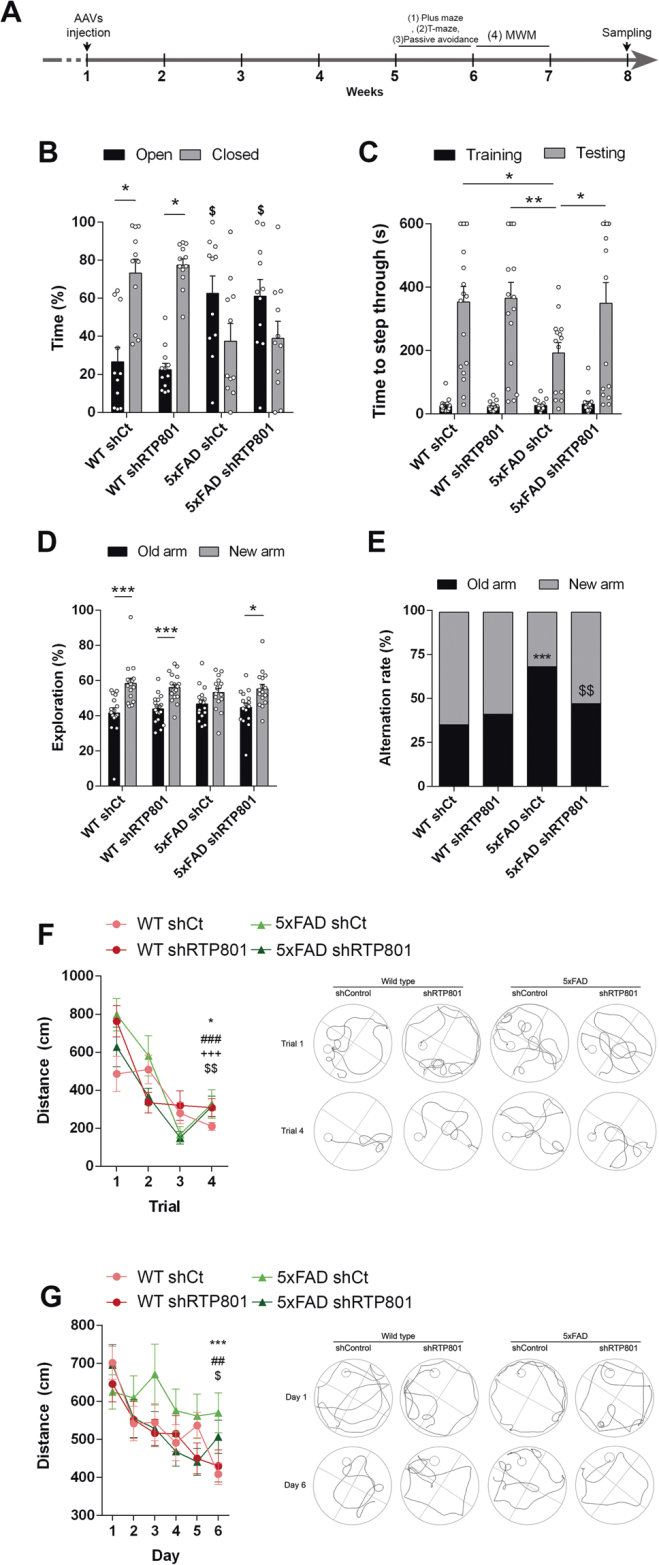
Behavioral phenotype upon genetic inhibition of RTP801 levels in the dorsal hippocampus of 5xFAD male mice. As depicted in the scheme (**A**), AAVs expressing GFP-shCT (AAV-shCt) or GFP-shRNA-RTP801 (AAV-shRTP801) were bilaterally injected in the dorsal hippocampus of 6-month-old WT males (WT shCt or WT shRTP801 groups) or 5xFAD males (5xFAD shCt or 5xFAD shRTP801 groups). Four weeks later, a battery of behavioral tests was performed. **B** Elevated plus maze. The time spent in the open arms was monitored for 5 min in 7-month-old WT shCt, WT shRTP801, 5xFAD shCt, and 5xFAD shRTP801 groups of mice. A genotype significant effect was detected (two-way ANOVA, *F*_(1, 82)_ = 6.633, *P* = 0.0118). **C** Passive avoidance test. The latency (in seconds) to step-through was measured before (Training) and 24 h after (Testing) receiving an electric shock (2 s 1 mA) in 7-month-old WT shCt, WT shRTP801, 5xFAD shCt, and 5xFAD shRTP801 groups of mice. A significant effect by groups was detected (two-way ANOVA, *F*_(1,126)_ = 126.9, *P* = 0.0001). **D**, **E** Spontaneous alternation learning in a T-maze. Spontaneous alternation learning was assessed by the T-SAT in 7-month-old WT shCt, WT shRTP801, 5xFAD shCt, and 5xFAD shRTP801 groups of mice for arms exploration (Arm preference effect: *F*(_1, 126)_ = 45.26, *P* < 0.0001) and for spontaneous alternation rate (WT shCt vs 5xFAD shCt: χ^2^:_22.04_, *P* < 0.0001 and 5xFAD shCt vs 5xFAD shRTP801: *χ*^2^:_9.148_, *P* < 0.01) 1 h after the training trial. **F**, **G** Morris water maze. **F** The distance (in cm) to reach the visible platform was monitored in a 4-trials session to evaluate potential visual or physical impairments in 7-month-old WT shCt, WT shRTP801, 5xFAD shCt, and 5xFAD shRTP801 groups of mice. Two-way ANOVA showed a significant general time effect in this procedural version of the MWM (*F*_(3,192) _= 35.12, *P* < 0.0001) and post hoc (Tukey’s test) multiple comparisons indicated that all groups significantly improved their latencies in trial 4 compared to trial 1 (see graph). **G** The distance (in cm) to reach the hidden platform was monitored in a daily 4-trials session performed for 6 days to evaluate spatial learning in 7-month-old WT shCt, WT shRTP801, 5xFAD shCt, and 5xFAD shRTP801 groups of mice. Two-way ANOVA showed a significant general time effect in this spatial version of the MWM (*F*_(5,320)_ = 9.413, *P* < 0.0001) and post hoc (Tukey’s test) multiple comparisons indicated that all groups significantly improved their latencies to reach the hidden platform on the day of training 6 compared to the day of training 1, except the 5xFAD shCt group who showed no significant differences comparing its latencies on day 6 with those on day 1 (see graph). The number of mice in **F**, **G**, WT shCt (*n* = 16), WT shRTP801 (*n* = 18), 5xFAD shCt (*n* = 16), and 5xFAD shRTP801 (*n* = 16). Data are means ± SEM. In **B**, **C**, and **D**, a two-way ANOVA with Bonferroni’s post hoc test was performed: *P < 0.05, **P < 0.01, ***P < 0.001 when comparing open vs closed or training vs testing or old arm vs new arm on each genotype. In **B**, ^$^P < 0.05 vs. WT shCt open arm. In **E**, Chi-square (*χ*^2^) test was performed in pair comparisons: ***P < 0.001 compared with WT shCt and ^$$^*P* < 0.01 compared with 5xFAD shCt. In **F**, *P < 0.05 comparing trials 1 vs 4 in WT shCt, ^###^*P* < 0.001 comparing trials 1 vs 4 in WT shRTP801, ^+++^*P* < 0.001 comparing trials 1 vs 4 in 5xFAD shCt and ^$$^*P* < 0.01 comparing trials 1 vs 4 in 5xFAD shRTP801. In **G**, ***P < 0.001 comparing days 1 vs 6 in WT shCt, ^##^*P* < 0.01 comparing days 1 vs 6 in WT shRTP801 and ^$^*P* < 0.05 comparing days 1 vs 6 in 5xFAD shRTP801.

Four weeks after injection, we performed a broad behavioral characterization as depicted in Fig. 2A. First, all mice were subjected to the plus-maze paradigm since this test is sensitive to several neurological disturbances in 5xFAD mice at this age [34, 35]. In this test, the increased time spent in the open arms showed by 5xFAD shCt mice compared with WT shCt mice was not corrected in 5xFAD shRTP801 mice (Fig. 2B). In the passive avoidance test, however, the 5xFAD shCt mice showed significantly lower latencies to step-through in the testing session compared with WT shCt mice (Fig. 2C). Interestingly, these differences were completely rescued in the 5xFAD shRTP801 mice (Fig. 2C). In the spontaneous alternation in a T-maze task (Fig. 2D, E), the arm preference and spontaneous alternation were evaluated 1 h after habituation. We found that the WT shCt, WT shRTP801, and 5xFAD shRTP801 mice explored significantly longer the novel arm than the familiar one whereas 5xFAD shCt mice showed no preference for any arm (Fig. 2D). Furthermore, WT shCt, WT shRTP801, and 5xFAD shRTP801 mice significantly alternated whereas 5xFAD shCt mice did not (Fig. 2E). Thus, in the T-SAT 5xFAD shCt mice showed alterations in both variables, spontaneous alternation, and new context exploration, whereas 5xFAD shRTP801 mice showed significant restoration of these two parameters. Finally, we used the Morris water maze (MWM) to test possible alterations in associative and spatial learning. First, all groups showed normal muscular strength in the grid test (WT shCt 60.0 ± 0.0, 5xFAD shCt 53.36 ± 3.636, WT shRTP801 60.0 ± 0.0 and 5xFAD shRTP801 60.0 ± 0.0; two-way ANOVA genotype effect: *F*_(1,41)_ = 1.047, *P* = 0.3122; two-way ANOVA shRNA effect: *F*_(1,41)_ = 1.047, *P* = 0.3122), suggesting the idea that potential changes in the MWM are not due to muscular strength deficits. Second, we found no differences in mice weight associated to RTP801 silencing although 5xFAD mice presented decreased body weight, as previously reported [36] (WT shCt 31.11 ± 2.11, 5xFAD shCt 29.64 ± 2.798, WT shRTP801 31.09 ± 3.093, and 5xFAD shRTP801 29.10 ± 2.935; two-way ANOVA treatment effect: *F*_(1,62)_ = 0.1616, *P* = 0.6891; two-way ANOVA genotype effect: *F*_(1,62)_ = 6.358, *P* = 0.0143). Next, to exclude poor vision, altered motivation, and/or sensorimotor disabilities in 5xFAD groups of mice, all the animals were tested in the visible platform task (four trials per mouse). We found no differences between WT shCt, WT shRTP801, 5xFAD shCt, and 5xFAD shRTP801 mice in escape latencies during training with the visible platform (Fig. 2F). All the groups of mice improved rapidly across trials demonstrating that the task was acquired normally. When spatial learning was assessed using the hidden platform version of the MWM, we found that the performance improved significantly during training in WT shCt, WT shRTP801, and 5xFAD shRTP801 mice but not in 5xFAD shCt mice (Fig. 2G). Thus, WT shCt, WT shRTP801, and 5xFAD shRTP801 mice showed acquisition of spatial learning and memory, whereas 5xFAD shCt mice did not. Altogether, this set of results shows that 7.5-month-old 5xFAD mice suffer from deficits in associative and spatial learning and that the normalization of RTP801 levels in principal neurons of the CA1 and dentate gyrus (in the 5xFAD shRTP801 group) rescues these cognitive impairments.

### Genetic normalization of hippocampal RTP801 levels in 5xFAD mice corrects core neuroinflammatory events

**Fig. 3:**
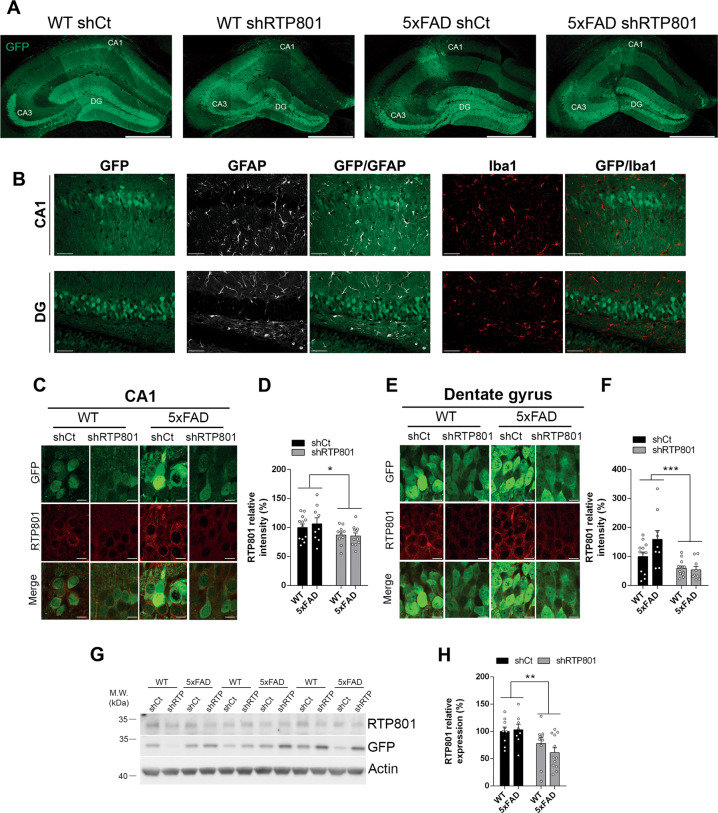
Genetic inhibition of RTP801 levels in the dorsal hippocampus of 5xFAD mice. **A** Representative dorsal hippocampi from WT shCt, WT shRTP801, 5xFAD shCt, and 5xFAD shRTP801 mice 8 weeks after the injection, GFP fluorescence (green) was detected in the entire dorsal hippocampus. **B** Neuronal transduction specificity with AAV 2/8. Representative CA1 and DG images from dorsal hippocampus where transduced cells (GFP + , green), astrocytes (GFAP + , white), and microglial cells (Iba1 + , red) are depicted. Transduced cells were found in the CA1 pyramidal layer and DG granular layer. No glial cells were transduced. **C** Representative immunostained CA1 pyramidal cells stained for GFP (green) and RTP801 (red). **D** Immunoreactivity quantification of the RTP801 levels in pyramidal neurons of the CA1 in the four groups is shown (treatment effect: *F*_(1, 38)_ = 6.197, *P* = 0.0173). **E** Representative immunostained granular cells of the DG stained for GFP (green) and RTP801 (red). **F** Immunoreactivity quantification of the RTP801 levels in granular neurons in the four groups is shown (treatment effect: *F*_(1, 38)_ = 17.73, *P* = 0.0002). **G** Representative immunoblots showing the levels of RTP801 relativized with respect to GFP/actin ratio levels as the corresponding loading controls in dorsal hippocampus extracts from WT shCt, WT shRTP801, 5xFAD shCt, and 5xFAD shRTP801 groups of mice. **H** The histogram represents the protein expression expressed as a percentage of WT shCt (treatment effect: *F*_(1,35)_ = 11.00, *P* = 0.0021). In (**G**), molecular weight markers position is indicated in kDa. All data are shown as the mean ± SEM. All data were analyzed by two-way ANOVA followed by Bonferroni’s post hoc test: *P < 0.05, **P < 0.01, ***P < 0.001. DG dentate gyrus. Scale bars: **A** 500 microns; **B** 50 microns; **D** 10 microns.

**Fig. 4:**
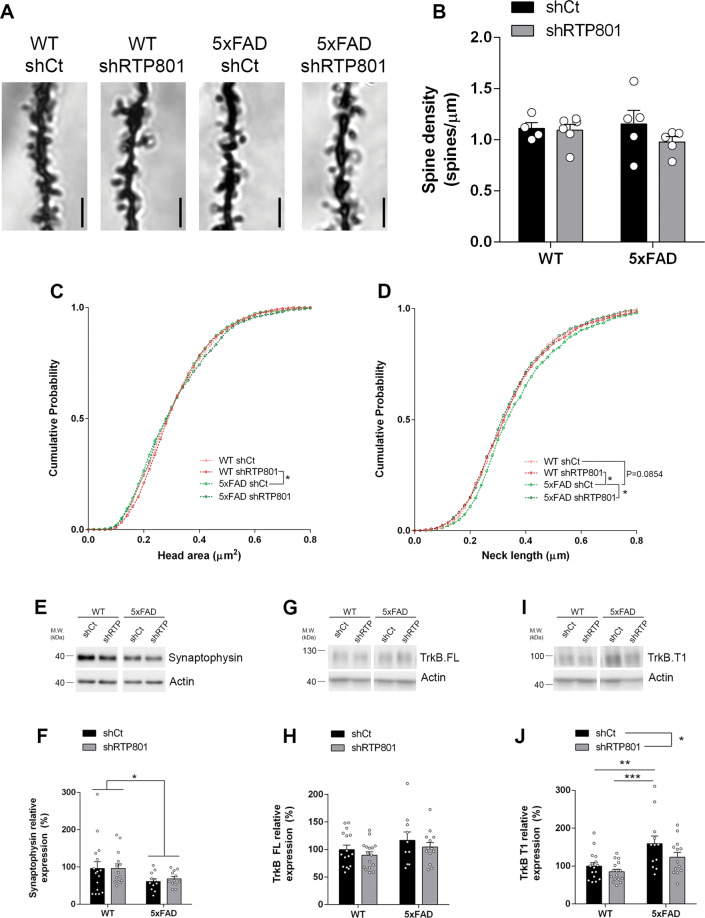
Spine density and morphology and synaptic markers in the dorsal hippocampus in shRTP801-injected mice. **A** Golgi–Cox-stained proximal apical dendrites of CA1 stratum radiatum pyramidal neurons. Scale bar, 2 µm. **B** Quantification of spine density in dendrites as in (**A**). Data show mean spine density from 20 dendrites per animal (two-way ANOVA treatment effect: *F*_(1,16)_ = 1.297, *P* = 0.2716). **C**, **D** Cumulative probability of spine head area (**C**) and spine neck length (**D**) in 10–15 dendrites per animal from four to six animals per group. Distributions were compared with the Kolmogorov–Smirnov test. *P < 0.05. No differences were obtained in spine head diameter. Neck length, WT shCt vs. 5xFAD shCt, D = 0.06287 *P* = 0.0854; WT shRTP801 vs. 5xFAD shCt, D = 0.07492, *P* = 0.0224; 5xFAD shCt vs. 5xFAD shRTP801,D = 0.07158, *P* = 0.0467. **E**–**J** Immunoblotting for the synaptic markers synaptophysin (**E**), TrkB.FL (**G**) and TrkB.T1 (**I**) and actin as the loading control. Densitometric quantification of synaptophysin (**F**) (genotype effect: *F*_(1,49)_ = 5.538, *P* = 0.0227), TrkB.FL (**H**) (treatment effect: *F*_(1,51)_ = 1.558, *P* = 0.2176) and TrkB.T1 (**J**) (treatment effect: *F*_(1,53)_ = 4.281, *P* = 0.0434) as in (**E**, **G**, and **I**). In (**E**, **G**, and **I**) molecular weight markers position is indicated in kDa. All data are shown as the mean ± SEM. All data were analyzed by two-way ANOVA followed by Bonferroni’s post hoc test: *P < 0.05, **P < 0.01, ***P < 0.001.

One week after behavioral testing, we sacrificed the animals to investigate the key events that could explain memory and learning improvement. We first confirmed widespread viral transduction in the dorsal hippocampus including the dentate gyrus (DG), the CA3, and the CA1 (Fig. 3A) and specific transduction of neuronal cells (Fig. 3B). We then confirmed by immunohistochemistry that the RTP801 levels were downregulated by the shRTP801 in the pyramidal neurons of the CA1 (Fig. 3C, D) and in the granular neurons of the DG (Fig. 3E, F), and found a 15% and a 40% decrease, respectively. These changes were further confirmed by western blot by evaluating RTP801 and GFP protein levels in the whole dorsal hippocampus (Fig. 3G, H), where we observed a 30–40% decrease in RTP801 levels in shRTP801-injected mice. We next studied whether RTP801 silencing was affecting plaque load in the dorsal hippocampus but no changes were found between 5xFAD shCt and 5xFAD shRTP801 animals (Supplementary Fig. 2). Next, we investigated whether RTP801 downregulation was affecting synaptic plasticity. Golgi–Cox staining showed no differences in spine density between the four groups (Fig. 4A, B). No significant differences were observed in the size of the head area of these spines either (Fig. 4C). However, silencing RTP801 in 5xFAD animals rescued the excessive neck length of their dendritic spines (Fig. 4D). Regarding the levels of synaptic proteins, we observed that RTP801 expression normalization in the 5xFAD mice did not prevent the loss of synaptophysin, previously reported to be decreased in 5xFAD mice at this age [29, 35] (Fig. 4E, F). Levels of full-length TrkB receptor (TrkB.FL), another neuronal marker that participates in AD pathology [37], were not affected either (Fig. 4G, H). However, the levels of the truncated TrkB (TrkB.T1), an isoform of the receptor with important regulatory effects in astrocytes and associated with toxicity and inflammation [38–40], were corrected after knocking down RTP801 in the 5xFAD hippocampus (Fig. 4I, J).

**Fig. 5:**
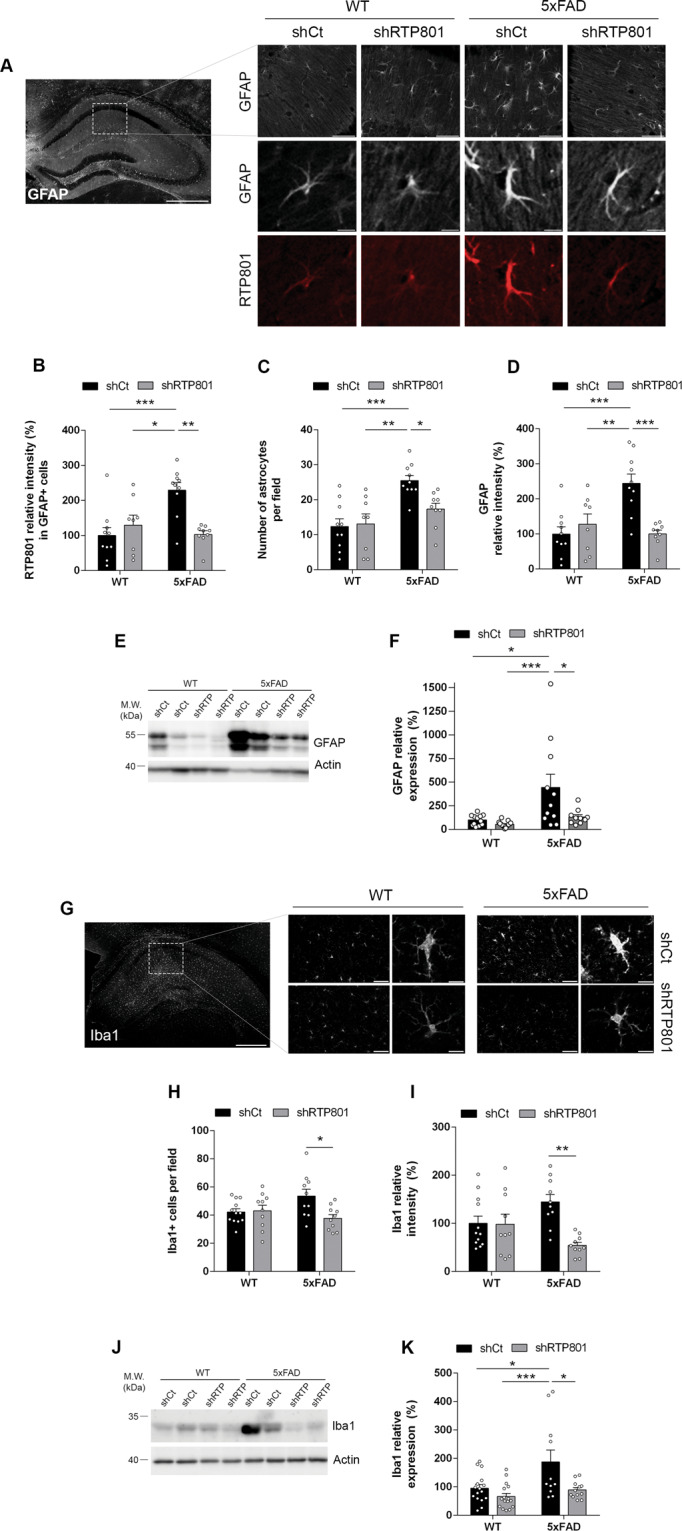
Genetic inhibition of hippocampal RTP801 levels and neuroinflammatory markers. **A** Representative GFAP immunofluorescence microscopy imaging in the dorsal hippocampus (left panel, scale bar, 500 microns) and representative GFAP and RTP801 labeling in inset images from the CA1 in 7.5-month-old WT shCt, WT shRTP801, 5xFAD shCt, and 5xFAD shRTP801 groups of mice (right panels, scale bars 100 microns and 10 microns, respectively). **B** Quantification of RTP801 levels (IOD intensity) in CA1 GFAP-positive cells from **a** in the four groups (treatment effect: *F*_(1, 33)_ = 4.995, *P* = 0.0323; interaction effect: *F*_(1, 33)_ = 13.11, *P* = 0.001). **C** Quantification of GFAP-positive cell density in the CA1 from **A** in the four groups (treatment effect: *F*_(1, 33)_ = 3.259, *P* = 0.0802; Interaction effect: *F*_(1, 33)_ = 4.653, *P* = 0.0384). **D** Quantification of GFAP relative intensity (% respect to WT shCt) in the CA1 from **A** in the four groups (treatment effect: *F*_(1, 33)_ = 6.445, *P* = 0.0160; interaction effect: *F*_(1, 33)_ = 14.11, *P* = 0.0007). **E** Immunoblotting for GFAP and actin as a loading control in the hippocampus of 7.5-month-old WT shCt, WT shRTP801, 5xFAD shCt, and 5xFAD shRTP801 groups of mice. **F** Densitometric quantification of GFAP results as in (**e**) for the hippocampus (treatment effect: *F*_(1, 44)_ = 9.941, *P* = 0.0029; genotype effect: *F*_(1, 44)_ = 7.085, *P* = 0.0108). **G** Representative Iba1 immunofluorescence microscopy imaging in the dorsal hippocampus (left panel, scale bar, 500 microns) and representative Iba1 labeling in inset images from the CA1 in 7.5-month-old WT shCt, WT shRTP801, 5xFAD shCt, and 5xFAD shRTP801 groups of mice (right panels, scale bars; 100 and 10 microns respectively). **H** Quantification of Iba1-positive cell density in the CA1 from **G** in the four groups (treatment effect: *F*_(1, 38)_ = 4.952, *P* = 0.0321; interaction effect: *F*_(1, 38)_ = 6.210, *P* = 0.0172). **I** Quantification of Iba1 relative intensity (% respect to WT shCt) in the CA1 from **G** in the four groups (treatment effect: *F*_(1, 38)_ = 8.821, *P* = 0.0051; interaction effect: *F*_(1, 38)_ = 8.211, *P* = 0.0067). **J** Immunoblotting for Iba1 and actin as a loading control in the hippocampus of 7.5-month-old WT shCt, WT shRTP801, 5xFAD shCt, and 5xFAD shRTP801 groups of mice. **K** Densitometric quantification of Iba1 results as in (**J**) for the hippocampus (treatment effect: *F*_(1, 51)_ = 10.16, *P* = 0.0024; genotype effect: *F*_(1, 51)_ = 8.448, *P* = 0.0054). Data are means ± SEM. In all panels, two-way ANOVA with Bonferroni’s post hoc test was performed: *P < 0.05, **P < 0.01, ***P < 0.001.

In line with that, we observed that RTP801 levels were also heavily upregulated in the astrocytes of the CA1 in 5xFAD mice compared to WT mice. Although astrocytes were not transduced with our neuron-specific AAVs serotype (Fig. 3B), knocking down RTP801 in 5xFAD mice reduced astroglial RTP801 levels similar to WT mice injected with shCt AAVs (Fig. 5A, B), suggesting an RTP801-mediated effect from neurons to glial cells in the 5xFAD mice. Reinforcing this idea, 5xFAD mice transduced with shCt displayed an increased number of astrocytes per field (Fig. 5A–C) and increased GFAP-immunoreactivity intensity (Fig. 5A–D) compared to WT shCt whereas 5xFAD mice transduced with shRTP801 AAVs showed a complete rescue of these two parameters (Fig. 5C, D). Finally, a similar rescue effect was observed by western blot evaluating total GFAP levels in the entire dorsal hippocampus (Fig. 5E, F) strengthening the idea that normalization of RTP801 levels in hippocampal principal neurons restores and ameliorates the astrogliosis processes associated with the 5xFAD mutations.

To further gain insight into the neuroinflammatory processes mediated by RTP801 in the 5xFAD mice, we then evaluated microglial processes in the same animals. Microgliosis or changes in microglial states have been described in neurodegenerative disorders such as AD [41]. To do so, we first assessed the number of Iba1-positive microglial cells in the CA1, which trended to increase in 5xFAD shCt mice, whereas in 5xFAD mice transduced with shRTP801 this number was significantly reduced (Fig. 5G, H). Similar results were observed when the intensity of Iba1-immunoreactivity was evaluated, showing a trend to increase in 5xFAD shCt and the same parameter was significantly reduced in 5xFAD mice transduced with shRTP801 (Fig. 5G–I). Finally, global changes on Iba1 levels in the entire dorsal hippocampus were also evaluated by western blot and we observed a significant increase of Iba1 protein levels in the 5xFAD shCt mice compared with WT shCt mice whereas this increase was completely rescued in 5xFAD shRTP801 mice (Fig. 5J, K).

We observed that the effect of silencing RTP801 in the 5xFAD mice was mTOR-independent (Supplementary Fig. 3) since we did not observe any changes in either the phosphorylation levels of mTOR or phospho-S6RP, as an mTORC1 kinase activity readout (Supplementary Fig. 3a–d). We only saw a significant increase of the levels of phospho-Ser473 Akt, as an mTORC2 substrate, in the 5xFAD animals that were not significantly ameliorated upon RTP801 silencing (Supplementary Fig. 3e, f).

**Fig. 6:**
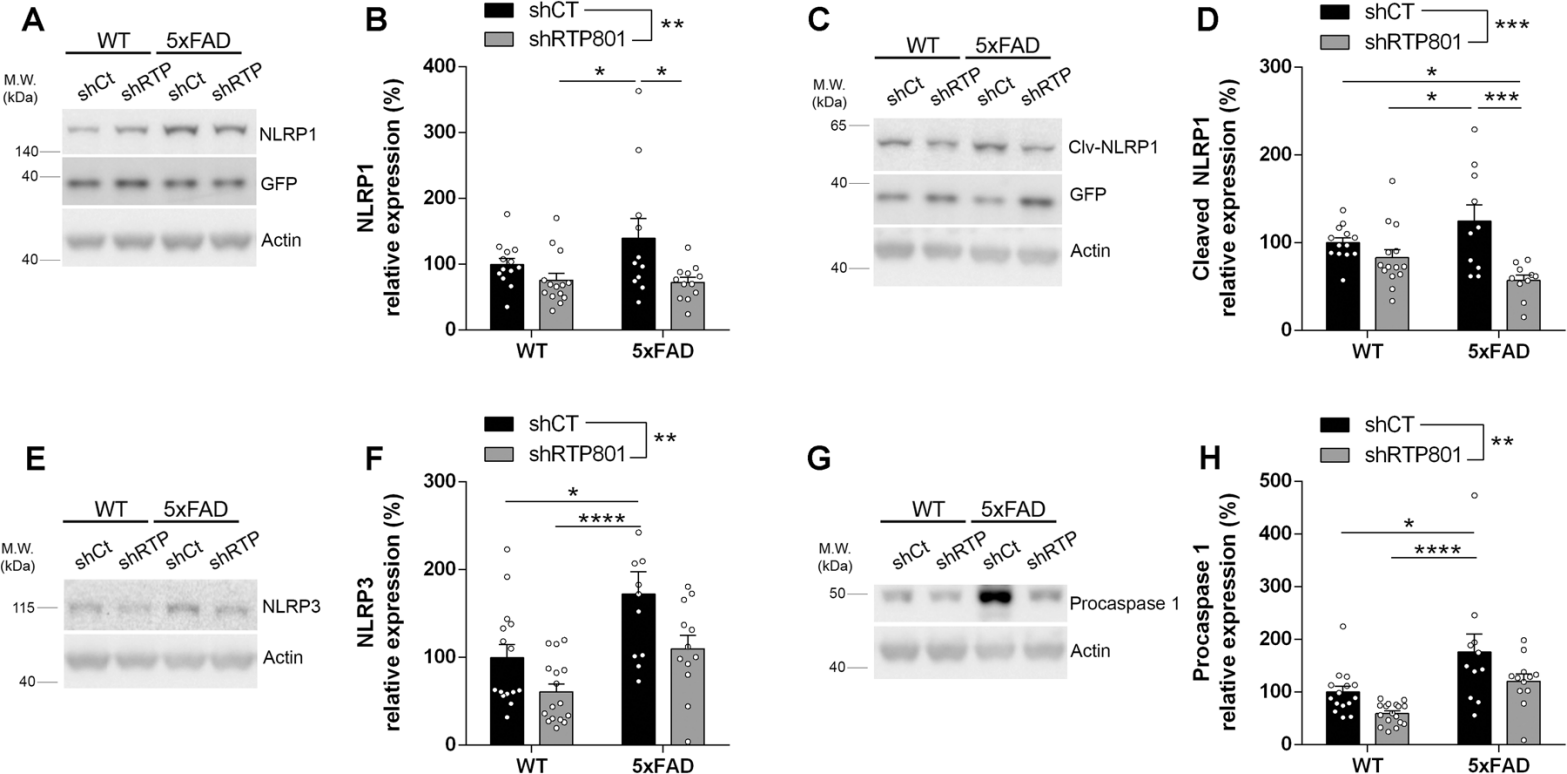
Genetic inhibition of RTP801 levels in the dorsal hippocampus reduces the levels of inflammasome components NLRP1 and NLRP3 and procaspase 1. **A**, **C** Immunoblottings for NLRP1, cleaved NLRP1, and GFP as loading control for transduced neurons in the dorsal hippocampus of 7.5-month-old WT shCt, WT shRTP801, 5xFAD shCt, and 5xFAD shRTP801 groups of mice. **B**, **D** Densitometric quantification of NRLP1 and cleaved NLRP1 results as in (**A**, **C**) for the hippocampus (NLRP1 treatment effect: *F*_(1, 46)_ = 8.714, *P* = 0.0050, interaction: *F*_(1, 46)_ = 1.932, *P* = 0.1713; cleaved NLRP1 treatment effect: *F*_(1, 46)_ = 15.84, *P* = 0.0003, interaction: *F*_(1, 46)_ = 5.702, *P* = 0.0214). **E**, **G** Immunoblottings for NLRP3 and procaspase 1 and actin as loading control in the dorsal hippocampus of 7.5-month-old WT shCt, WT shRTP801, 5xFAD shCt, and 5xFAD shRTP801 groups of mice. **F**, **H** Densitometric quantification of NRLP3 and procaspase 1 results as in (**E**, **G**) for the hippocampus (NLRP3 treatment effect: *F*_(1, 50)_ = 10.28, *P* = 0.0023, genotype effect: *F*_(1, 50)_ = 14.72, *P* = 0.0004; procaspase 1 treatment effect: *F*_(1, 51)_ = 8.438, *P* = 0.0054, genotype effect: *F*_(1, 51)_ = 17.29, *P* = 0.0001). Data are means ± SEM. In all panels two-way ANOVA with Bonferroni’s post hoc test was performed: *P < 0.05, **P < 0.01, ***P < 0.001.

Hence, to deepen the investigation of the mechanism by which silencing RTP801 in neurons diminished the inflammatory response in the 5xFAD model, we assessed the levels of inflammasome receptors and their components. We detected that elevated levels of NLRP1, which is mainly expressed in neurons [42], as pro-form (Fig. 6A, B) and auto-proteolytic fragment (cleaved) (Fig. 6C, D), are normalized by RTP801 silencing in neurons. The same results were seen for NLRP3, a receptor generally expressed in astrocytes and microglia (Fig. 6E, F). Levels of procaspase 1, a common effector for NLRP1 and 3, were also normalized after silencing RTP801 in neurons (Fig. 6G, H). On the contrary, we did not observe significant changes in cleaved caspase 1 (p20) levels or IL-1β production (Supplementary Fig. 4a–e). AIM2 levels, another inflammasome receptor activated by DNA damage, were sensitive to RTP801 silencing (Supplementary Fig. 4d, f).

Altogether, our results suggest that silencing RTP801 levels in hippocampal principal neurons induces, in turn, a correction of aberrantly increased levels of RTP801 in astrocytes and significant amelioration of general neuroinflammatory processes in 5xFAD mice by regulating the NLRP1 and NLRP3 inflammasomes.

## Discussion

Here, we found that the stress-induced protein RTP801 is upregulated in the hippocampus from both human AD patients and in the 5xFAD and tauopathy murine models of AD. Moreover, RTP801 levels in human postmortem hippocampal samples correlated with Braak and Thal stages that classify disease progression and severity. Remarkably, in these samples, RTP801 levels also correlated with GFAP expression, as a marker of astrogliosis. Indeed, RTP801 expression abrogation in the 5xFAD hippocampus prevented the cognitive decline associated with Aβ deposition. Interestingly, RTP801 downregulation was associated with an anti-inflammatory effect with a dramatic decrease of astrogliosis, microgliosis, and a reduction of AIM2, NLRP1, and NLRP3 inflammasome sensor proteins.

In human postmortem hippocampal samples, we observed that RTP801 levels were significantly elevated in whole-brain lysates and the synaptic compartment. This is in line with previous results in other neurodegenerative diseases such as PD [14, 16] and HD [17, 19], supporting the crucial role of RTP801 in neurodegeneration. Moreover, the correlation of RTP801 levels with the Braak and the Thal stages suggests that RTP801 could be considered as a biomarker in AD. Since Damjanac et al. also found RTP801 mRNA and protein levels elevated in lymphocytes derived from AD patients [24], we speculate that RTP801 could be treated as a systemic responsive protein in AD pathology and/or progression.

We found that RTP801 was elevated in compromised structures of the 5xFAD model and the rTg4510 tauopathy mouse models, in the hippocampus and the entorhinal cortex, respectively. This result suggests that the upregulation of RTP801 is common in the Aβ and the phospho-Tau toxic signaling cascades, in line with the positive correlation that we observed between RTP801 levels and Thal and Braak stages. Indeed, RTP801 was differentially elevated in the crude synaptosomal compartment from both transgenic models, suggesting a specific dysregulation of RTP801 at the synapses. RTP801 has been recently described to be significantly elevated in human HD and R6/1 mice striatal synaptic preparations [19]. These results suggest that altered synaptic RTP801 levels could be a common molecular mechanism in neurodegenerative diseases.

5xFAD mice display severe impairments in associative and spatial learning [35, 43] as occurs in human patients with AD [44–47]. Behavioral testing confirmed a remarkable rescue of cognitive skills by specifically silencing RTP801 in principal neurons (pyramidal and granular neurons) of the 5xFAD hippocampus. Hence, RTP801 mediates the loss of associative and declarative memories. However, this rescue did not involve changes in plaque load and size, suggesting that RTP801 function in AD does not modulate Aβ homeostasis.

The plastic role of RTP801 was first described by Ota and colleagues in a model of depression induced by chronic unpredicted stress, where RTP801 knockout mice were more resilient to stress and showed much less dendritic spine loss [20]. A similar role was corroborated in an A53T α-synuclein mouse model of PD under chronic restraint stress [18]. Recently, we found that RTP801-mediated motor-learning deficits in the R6/1 mouse model of HD [19].

Strikingly, RTP801 knockdown did not affect spine density but prevented the excessive spine neck length found in the 5xFAD. Longer spine necks have been associated with a decrease in the amplitude in EPSCs recordings, while shorter spine necks are associated with increased synaptic strength [48–50]. Hence, this morphological feature due to RTP801 silencing in the 5xFAD could be contributing to the prevention-cognitive deficits in this model.

The implication of RTP801 in the activation of inflammatory pathways has been addressed in several in vitro and in vivo studies [51–54]. However, the putative role of the protein in the inflammatory pathways in the nervous system had never been studied. Here, we found that RTP801 silencing reduced TrkB-truncated receptor isoform t1 (TrkB.T1). TrkB.T1 is present in senile plaques, elevated in AD brain samples and its overexpression in a mouse model of AD aggravates its memory impairment [38, 55, 56]. Interestingly, TrkB.T1 is the only TrkB isoform expressed by astrocytes [57] and regulates gliosis and the inflammatory response [58, 59]. Hence, these results suggest that astroglia response could be involved in the RTP801-dependent cognitive alterations in the 5xFAD model.

Reinforcing this, genetic RTP801 inhibition in hippocampal 5xFAD neurons had a general anti-inflammatory response, since it normalized in astrocytes the higher levels of GFAP but also the levels of RTP801 in comparison to WT. Although RTP801 expression in human and murine astrocytes has been described [60], its putative function in this cell type has never been investigated.

Astrocytes have emerged as key regulators of remote memory formation [61, 62] and molecular modifications in astroglial cells induce changes in rodent models of learning and memory [63]. Moreover, astrocytes are essential for the expression of synaptic plasticity phenomena such as long-term potentiation [64], displaying higher motility rates of their processes than dendritic spines [65]. Importantly, gliosis per se is strongly associated with cognitive decline [66, 67]. Hence, the correction of neuroinflammatory events upon genetic inhibition of neuronal RTP801 could be enough to observe significant memory improvements in the 5xFAD model.

A major question from our results is how transducing hippocampal principal neurons corrects astrogliosis and microgliosis in the 5xFAD mice since it seems to be mTOR-independent. We found that silencing neuronal RTP801 in 5xFAD mice downregulated the levels of two inflammasome receptors, NLRP1 and NLRP3, and their effector, procaspase 1. This lines with the results obtained in macrophages, adipocytes, and macrophage-adipocyte cocultures, where silencing RTP801/REDD1 diminished caspase 1 and NLRP3 levels as well as IL-1β secretion [53].

Since NLRP1 is mostly expressed in neurons [42], where the knockdown of RTP801 takes place and is sensitive to Aβ [68], we speculate that neuronal RTP801 silencing reduces NLRP1 inflammasome which, in turn, would contribute to preventing the NLRP3-mediated inflammatory response in glia and the increase of RTP801 levels in astrocytes.

The lack of significant differences in IL-1β or cleaved caspase 1 suggests that RTP801 silencing could be affecting the priming activation step, which depends on gene expression of NLRP1, NLRP3 and the procaspase 1 [69, 70]. On the other hand, procaspase 1 complexed with NLRPs in the absence of ASC can mediate other responses such as cell death [71]. This could become important in older 5xFAD mice where neuron death becomes evident (9–10 months old) [29], since silencing RTP801 seems to modulate the levels of procaspase 1.

More studies are warranted to understand the inflammatory crosstalk between neurons, microglia, and astrocytes over the timeline of the disease and how Aβ-induced neuronal RTP801 upregulation affects the other two cell types.

The advantage to target RTP801 levels, unlike mTOR activity, is that they are commonly upregulated in neurodegenerative conditions [17, 19, 72]. Therefore, its modulation could be more effective in a pathologic context of AD in comparison to targeting mTOR, with a wider and more complex spectrum of functions during the disease [73–75].

In summary, RTP801 is upregulated in AD mouse models and AD brains, and normalizing its hippocampal expression in the 5xFAD model prevented the appearance of the inflammatory response and restored cognitive deficits. This work frames RTP801 as a promising biomarker and a new pharmacological target in AD.

## Materials and methods

### Human postmortem samples

Postmortem hippocampal samples from Alzheimer’s disease patients and controls were obtained from Banc de Teixits Neurològics (IDIBAPS, Barcelona, Spain). The donation and obtaining of samples were regulated by the ethics committee of the Universitat de Barcelona. The sample processing followed the rules of the European Consortium of Nervous Tissues: BrainNet Europe II (BNEII). All the samples were protected in terms of individual donor identification following the BNEII laws. Case information can be found in Supplementary Table 1. All the procedures for the obtention of postmortem samples followed the ethical guidelines of the Declaration of Helsinki and local ethical committees (Universitat de Barcelona ethical committee: IRB00003099).

### Animal models

For this study, we used the transgenic mouse line 5xFAD (MMRRC catalog #034840-JAX, RRID:MMRRC_034840-JAX). 5xFAD mice overexpress the 695-amino acid isoform of the human amyloid precursor protein (APP695) carrying the Swedish, London, and Florida mutations under the control of the murine Thy-1 promoter. Besides, they express human presenilin-1 (PSEN-1) carrying the M146L/L286V mutation, also under the control of the murine Thy-1 promoter [29]. We also used for biochemical studies male and female transgenic mice from the line CtTA/rTg4510 (IMSR Cat# JAX:015815, RRID:IMSR_JAX:015815) expressing the P301L mutant variant of human four-repeat microtubule-associated protein Tau (0N4R tauP301L) at 6 months of age [33]. All animals were housed with access to food and water ad libitum in a colony room kept at 19–22 °C and 40–60% humidity, under a 12:12-h light/dark cycle. Experimental animals were all males and used at 6 months of age and following the ethical guidelines (Declaration of Helsinki and NIH Publication no. 85-23, revised 1985, European Community Guidelines, and Spanish guidelines (RD53/2013) for handling animals and approved by the local ethical committee (University of Barcelona, 225/17 and Generalitat de Catalunya, 404/18).

### Tissue fixation and immunofluorescence

Animals were euthanized by cervical dislocation. Left hemispheres were removed and fixed for 5 days in 4% paraformaldehyde in PBS. Free-floating coronal brain sections (40 µm) were obtained using a Leica vibratome (Leica VT1000S). Sections were first washed twice in PBS-T (1× PBS 0.3% Triton X-100) and incubated in 50 mM NH_4_Cl, twice for 15 min. Blocking and permeabilization were performed for 1 h in PBS-T with 0.02% azide, 3% NGS, and 0.2% BSA. For amyloid plaque staining, blocking was performed for 4 h, and blocking buffer contained 10% donkey serum in PBS 0.25% Triton X-100. Primary antibodies were diluted in blocking solution and incubated overnight at 4 °C in agitation. Secondary antibodies were diluted in blocking buffer and incubated for 2 h at room temperature. Nuclei were stained with Hoechst33342 (Thermo Fisher Scientific, #H3570) diluted 1:5000 in PBS for 15 min. Sections were washed with PBS-T between the different steps, and a final wash with PBS was performed prior to mounting with ProLong Gold Antifade Mountant. The following primary antibodies were used: anti-GFP chicken polyclonal (1:1000, Synaptic systems, #132006), anti-GFAP mouse monoclonal (1:1000, Sigma, #G3893), anti-GFAP rabbit polyclonal (1:500, Dako, #GA52461), anti-Iba1 rabbit polyclonal (1:500, Wako, #09-19741), anti-RTP801 rabbit polyclonal (1:100, Proteintech, #10638-1-AP) and anti-APP (1:1000, Novus Biologicals, #NBP2-62566). The following secondary antibodies were used: goat anti-chicken AlexaFluor488 (1:500, #A11039), goat anti-mouse AlexaFluor555 (1:200, #A21424), goat anti-mouse AlexaFluor647 (1:200, #A21236), goat anti-rabbit AlexaFluor555 (1:200, #A21429), goat anti-rabbit AlexaFluor647 (1:200, #A21245) and donkey anti-rabbit AlexaFluor555 (1:600, #A32794) (all from Thermo Fisher Scientific) (see Supplementary Table 2). Thioflavin S (ThioS) staining was performed in free-floating sections following immunofluorescence procedure for APP and was performed as described in ref. [76]. Images were obtained with confocal microscopy (Zeiss LSM 880 and ZEN Software) at the Microscopy Service (Campus Clínic) with a ×10, ×25, or ×40 magnification and standard (1 airy disc) pinhole (1 AU). Two sections from the dorsal hippocampus were analyzed per animal.

### Crude synaptosomal fractionation

Crude synaptosomal fractions were isolated from both murine and human hippocampal brain samples. Samples were first homogenized in Krebs–Ringer (KR) buffer (125 mM NaCl, 1.2 mM KCl, 22 mM NaHCO_3_, 1 mM NaH_2_PO_4_, 1.2 mM MgSO_4_,1.2 mM CaCl_2_, pH 7.4) supplemented with 10 mM glucose, 0.31 M sucrose (Sigma), and protease and phosphatase inhibitors (PhosSTOP and cOmplete, both from Roche, and PMSF, 1:100, from Sigma). Samples were centrifuged at 1.000 × *g* for 10 min to discard debris. A sample of the resulting supernatant was kept as the Homogenate fraction. Next, the supernatant was centrifuged at 16.000 × *g* for 15 min to obtain the cytosolic fraction and the crude synaptosomal fraction (resuspended in supplemented KR buffer).

### Western blotting

Animals were euthanized by cervical dislocation. For western blot analyses, both hippocampi or entorhinal cortex were dissected out and stored at −80 °C until use. For AAV-injected 5xFAD mice, the dorsal and ventral hippocampus were dissected out separately. Samples (15–20 µg) were resolved with NuPAGE^TM^Novex^TM^ polyacrylamide gels (3–8% polyacrylamide gels with Tris-Acetate running buffer were used to analyze proteins with high molecular weight, while 12% and 4–12% polyacrylamide gels with MOPS SDS running buffer were used for proteins with small and intermediate weights, respectively). Proteins were transferred to nitrocellulose membranes with the iBlot2 system. All reagents and machinery were obtained from Thermo Fisher Scientific. Membranes were blocked with 5% non-fat dry milk (Biorad) diluted in TBS-T (Tris-buffered saline containing 0.1% Tween-20). Primary antibodies were diluted in TBS-T with 5% BSA (Sigma) and incubated overnight at 4 °C by shaking. The following primary antibodies were used (1:1.000 if not stated otherwise): anti-GFAP (Dako, #GA52461), anti-GFP (Thermo Fisher Scientific, #A-11122), anti-RTP801 (1:500, Proteintech, #10638-1-AP), anti-Iba1 (Wako, #019-19741), anti-Synaptophysin (Synaptic Systems, #101011), anti-TrkB (BD Biosciences, #610102), anti-SV2a (Santa Cruz Biotech Technology, #sc-376234), anti-P-Akt Ser473 (Cell Signaling Technologies, #4691), anti-Total Akt (Cell Signaling Technologies, #4691), anti-P-S6 Ser235/236 ((Cell Signaling Technologies, #4858), anti-Total S6 (Cell Signaling Technologies, #2317), anti-P-mTOR Ser2448 (Cell Signaling Technologies, #2971), anti-Total mTOR (Cell Signaling Technologies, #2972), anti-NLRP1 (Novus, #NBP1-54899), anti-NLRP3, anti-procaspase 1, anti-cleaved caspase 1, anti-cleaved IL-1β, anti-ASC-TM1, anti-AIM2 (all from Cell Signaling Technologies, #20836T), and anti-β-actin (Sigma, #A3854). Mouse anti-actin primary antibody was already conjugated to horseradish peroxidase so it was incubated for 30 min before chemiluminescent protein detection. Anti-mouse and anti-rabbit secondary antibodies produced in goat and conjugated to HRP (Thermo Fisher Scientific, #31460 and #31430) were diluted 1:10.000 in blocking solution for 1 h at room temperature (summarized in Supplementary Table 3). Proteins were detected with Supersignal^TM^ West Pico Plus chemiluminescent substrate (Thermo Fisher Scientific). Images were acquired with ChemiDoc^TM^ (Bio-Rad) and quantified by densitometric analysis with ImageJ software (NIH). When reincubation with another primary antibody was needed, membranes were washed with Restore Plus Western Blot Stripping buffer (Thermo Fisher Scientific) for 15 min to remove the previous signal.

### Behavioral tests

#### Plus maze

The apparatus was made with two opposing 30 × 8 cm open arms, and two opposing 30 × 8 cm arms enclosed by 15 cm-high walls placed 50 cm above the floor and dimly lit (60 lx). Each mouse was placed in the central square, facing an open arm, and the time spent in the open arms, which normally correlates with low levels of anxiety, was measured for 5 min.

#### Passive avoidance

The passive avoidance (light–dark) paradigm was conducted in a 2-compartment box, where 1 compartment was dimly lit (20 lx) and the other brightly lit (200 lx). Both chambers were connected by a door (5 × 5 cm). During training, mice were placed into the aversive brightly lit compartment, and upon entry into the preferred dimly lit compartment (with all four paws inside the dark chamber), they received a mild foot shock (2-s foot shock, 1 mA intensity). The latency of mice to enter into the dark chamber was recorded. Twenty seconds after receiving the foot shock, mice were returned to the home cage until testing 24 h later (long-term memory). For this retention test, mice were returned to the brightly lit compartment and the latency to enter the shock-paired compartment (dark chamber) was measured (10-min time cutoff).

#### Spontaneous alternation in a T-maze

The T-maze apparatus used was a wooden maze consisting of three arms, two of them situated at 180° from each other, and the third representing the stem arm of the T, situated at 90° to the other two. All arms were 45 cm long, 8 cm wide, and enclosed by a 20-cm wall. The maze was thoroughly painted with waterproof gray paint. Light intensity was 5 lux throughout the maze. A starting area (10 cm) was located at the end of the stem arm and closed by a wooden guillotine door. Two identical guillotine doors were placed in the entry of the arms situated at 180°. The maze was elevated 60 cm above the floor. In the training trial, 1 arm was closed (novel arm) and mice were placed in the stem arm of the T (home arm) and allowed to explore this arm and the other available arm (familiar arm) for 10 min, after which they were returned to the home cage. After 1 h (retaining test session), mice were placed in the stem arm of the T-maze and allowed to freely explore all three arms for 5 min. The first choice to turn either to the familiar arm or to the new arm (alternation rate, %) was monitored. In addition, the arm preference was also monitored and determined by calculation of the distance traveled in each arm × 100/total distance traveled in both arms (familiar and novel).

#### Morris water maze

Spatial learning was assessed using a mouse-adapted Morris water maze (MWM) [77]. In a first phase to discard visual/physical deficiencies, each mouse underwent four trials in the water maze (circular pool; diameter: 100 cm; height: 40 cm, water depth: 25 cm), with the visible platform and without extra maze distal cues. The escape platform (10 cm diameter) was made visible by the attachment of a high-contrast striped flag. In the second phase (learning/acquisition phase), animals were trained 6 days, four trials per day. Four positions around the edge of the tank were arbitrarily designated north (N), south (S), east (E), and west (W) to provide four alternative start positions and define the division of the tank into four quadrants: NE, SE, SW, and NW. The platform was then submerged 1 cm below the water surface and placed at the midpoint of one of the quadrants. Each mouse was allowed to swim until they located and climbed onto the submerged platform. Mice that failed to locate the platform after 60 s were removed from the water and placed on the platform. At the end of the trial, all mice were left on the platform for 15 s, before being returned to the home cage during the intertrial interval.

#### Grid test

For this test, mice were placed on a horizontal cage lid which was then agitated circularly three times and next turned upside down for 60 s 20 cm above a housing cage.

In all tasks, animal tracking and recording were performed using the automated SMART junior software (Panlab, Spain).

### Stereotaxic surgery and AAV transduction

Following anesthesia with a mixture of 2% oxygen and isoflurane (2% induction, 1.5% maintenance), we performed bilateral hippocampal injections of rAAV2/8-H1-shControl-RSV-GFP (1.2 × 10^13^ GCs), rAAV2/8-H1-shRTP801-RSV-GFP (1.07 × 10^13^ GCs) [19]. All the AAVs were purchased to the Unitat de Producció de Vectors from the Center of Animal Biotechnology and Gene Therapy at the Universitat Autònoma de Barcelona. We used the following coordinates (millimeters) from bregma (anteroposterior and lateral) and from the skull (dorsoventral); anteroposterior: −2.0; Lateral + /−1.5, and dorsoventral: −1.3 (CA1) and −2.1 (DG). The cannula was left to deliver 1 μl of 1:1 virus in each depth for 4 min, and two additional minutes were left to have complete virus diffusion. After 2 h of careful monitoring, mice were returned to their home cage for 3 weeks before starting the subsequent behavioral and biochemical analysis.

### Golgi staining and spine analysis

Fresh brain hemispheres were processed with the FD Rapid GolgiStainTM kit (FD Neurotechnologies) as described in [78]. In all, 100-µm sections were obtained in a cryostat and mounted in gelatin-coated superfrost coverslips prior staining procedure. Brightfield images of impregnated apical dendrites from dorsal hippocampal CA1 pyramidal neurons were captured with a Leica AF6000 microscope (×63 magnification and 1.6 Zoom). Stacks were taken every 0.2 µm and analyzed manually with FIJI software. Spine density was calculated in 20 proximal apical dendrites per animal, starting 5 µm apart from the ramification and in segments with no overlap with other branches. Spine density values were averaged to obtain the mean for each animal. For a more precise description of dendritic spine shape, spine head diameter, and spine neck length were measured with FIJI and were analyzed as a continuous distribution (1200 dendritic spines were analyzed for each group). Image acquisition and analysis were performed blindly.

### Statistics

Sample sizes were determined by using the power analysis method: 0.05 alpha value, 1 estimated sigma value, and 75% of power detection. All data are expressed as mean ± SEM. Normal distribution was tested with d’Agostino and Pearson omnibus normality test. If the test was passed, statistical analysis was performed using parametric statistical analysis. Before pairs of comparisons, we performed the F test to compare variances. In experiments with normal distribution statistical analyses were performed using the unpaired two-sided Student’s *t* test (95% confidence) and the two-way ANOVA with the Bonferroni’s or Tukey’s post hoc tests as appropriate and indicated in the figure legends. *T* test with Welch’s correction was applied when variances were unequal. Values of *P* < 0.05 were considered statistically significant. Correlation analyses were performed using Pearson. Kolmogorov–Smirnov test was used to analyze the distribution of dendritic spines’ shape. Grubbs and ROUT tests were performed to determine the significant outlier values. All experiments in this study were blinded and randomized by blocks of animals. All mice bred for the experiments were used for pre-planned experiments and randomized to experimental groups. Data were collected, processed, and analyzed randomly. The experimental design and handling of mice were identical across experiments. Littermates were used as controls with multiple litters (3–5) examined per experiment.

## Supplementary information

SUPP FIGURE LEGENDS

Supplementary TABLES

Supplementary Figure 1

Supplementary Figure 2

Supplementary Figure 3

Supplementary Figure 4
