# Supplementary material for: RTP801/REDD1 contributes to neuroinflammation severity and memory impairments in Alzheimer’s disease
Source: Cell Death Dis. 2021 Jun 15;12(6):616. doi: 10.1038/s41419-021-03899-y (PMC8206344; doi:10.1038/s41419-021-03899-y)
Supplement: Supplementary file 1 — SUPP FIGURE LEGENDS [file 41419_2021_3899_MOESM1_ESM.docx]

**SUPPLEMENTARY FIGURE LEGENDS:**

**Sup Fig 1. RTP801 and SV2a protein levels in the synaptosomal fraction from human post-mortem samples with Alzheimer’s disease.** (**a**) Immunoblotting for SV2a, RTP801 and Akt as a loading control in the crude synaptic fraction obtained from human post-mortem hippocampal samples from controls (CT) and Alzheimer’s disease (AD) patients. (**b**) Densitometric quantification of RTP801 results as in (**a**) for the hippocampus (t_18.04_=1.539, p = 0.1412). (**c**) Densitometric quantification of SV2a results as in (**a**) for the hippocampus (t_21_=2.790, p = 0.0110).

**Sup Fig 2. Amyloid load in 5xFAD shCt and 5xFAD shRTP801 mice’s dorsal hippocampus. (a)** Representative dorsal hippocampi from 5xFAD shCt and 5xFAD shRTP801 8 weeks after the injection. Amyloid plaques were stained with Thioflavin S (ThioS, green) and for APP (red). (**b-c**) APP+ and ThioS+ plaque density was evaluated in the CA1, CA3 and DG but no significative shRNA effect was found (Two-way ANOVA Treatment effect F_(1, 56)_=0.005087, p =0.9434 for APP+ plaques and F_(1, 57)_=3.717, p =0.0589 for ThioS+ plaques). (**d-e**) APP+ and ThioS+ plaque size was evaluated in the CA1, CA3 and DG but no significative shRNA effect was found (Two-way ANOVA Treatment effect F_(1, 53)_=0.2.855, p =0.0970 for APP+ plaques and F_(1, 56)_=0.6811, p =0.4127 for ThioS+ plaques). Scale bars: 250 and 100 microns, respectively.

**Sup. Fig. 3. mTOR activity in dorsal hippocampal samples from WT and 5xFAD mice.** (**a**, **c**, **e**) Immunoblotting for the mTOR pathway phosphorylated proteins mTOR at Ser2448 (a), mTORC1 readout S6 at Ser235/236 (c) and mTORC2 readout Akt at Ser473 (e) and total proteins mTOR, S6 and Akt as loading controls. (**b, d, f**) Densitometric quantification of phosphorylated proteins vs. Total levels is expressed as the mean ± SEM: P-mTOR S2448/mTOR (Treatment effect: F_(1, 54)_=2.308, p=0.1345), P-S6 S235/236/S6 (Treatment effect: F_(1, 31)_=0.9437, p=0.3389) and P-Akt S473/Akt (Treatment effect: F_(1, 53)_=0.9762, p=0.3276; Genotype effect: F_(1, 53)_=16.65, p=0.0002). All data were analyzed by two-way ANOVA followed by Bonferroni’s *post hoc* test: ** *P*<0.01.

**Sup. Fig. 4. Inflammatory molecules in the dorsal hippocampus from WT and 5xFAD mice.** (a, d) Immunoblottings for cleaved caspase 1, cleaved IL-1β, ASC-TM1, AIM2 and actin as loading control in the dorsal hippocampus of 7.5-month-old WT shCt, WT shRTP801, 5xFAD shCt and 5xFAD shRTP801 groups of mice. (b, c, e, f) Densitometric quantification of cleaved caspase 1 (b) (Treatment effect: F_(1, 50)_=0.3752, p=0.5429), cleaved IL-1β (Treatment effect: F_(1, 48)_=0.1296, p=0.7204), ASC-TM1 (Treatment effect: F_(1, 51)_=3.656, p=0.0615) and AIM2 (Treatment effect: F_(1, 51)_=4.208, p=0.0454). All data were analyzed by two-way ANOVA followed by Bonferroni’s *post hoc* test: ** *P*<0.01.
