# Supplementary material for: RTP801/REDD1 contributes to neuroinflammation severity and memory impairments in Alzheimer’s disease
Source: Cell Death Dis. 2021 Jun 15;12(6):616. doi: 10.1038/s41419-021-03899-y (PMC8206344; doi:10.1038/s41419-021-03899-y)
Supplement: Supplementary file 2 — Supplementary TABLES [file 41419_2021_3899_MOESM2_ESM.docx]

| **Patient** | **Clinical diagnosis** | **Braak stage** | **Thal stage** | **Sex** | **Age (years)** | **PMD (hh:mm)** |
| --- | --- | --- | --- | --- | --- | --- |
| 1 | Control | 3 | 0 | M | 86 | 7:25 |
| 2 | Control | 3 | 3 | F | 90 | 13:40 |
| 3 | Control | - | - | M | 58 | 05:00 |
| 4 | Control | 2 | 3 | F | 88 | 24:00 |
| 5 | Control | 5 | 5 | M | 78 | 05:00 |
| 6 | Control | 2 | 0 | F | 83 | 07:30 |
| 7 | Control | 2 | 5 | F | 97 | 07:20 |
| 8 | Control | 2 | 4 | F | 93 | 05:30 |
| 9 | Control | 2 | 5 | F | 83 | 07:33 |
| 10 | Control | 2 | 3 | M | 86 | 07:35 |
| 11 | AD | 6 | 5 | F | 85 | 12:00 |
| 12 | AD | 6 | 5 | F | 84 | 11:00 |
| 13 | AD | 6 | 4 | F | 80 | 15:00 |
| 14 | AD | 5 | 3 | M | 86 | 17:30 |
| 15 | AD | 6 | 5 | M | 85 | 15:35 |
| 16 | AD | 6 | 5 | F | 82 | 16:45 |
| 17 | AD | 6 | 5 | F | 78 | 11:30 |
| 18 | AD | 6 | 5 | M | 86 | 12:00 |
| 19 | AD | 6 | 5 | M | 78 | 07:20 |
| 20 | AD | 6 | 5 | F | 90 | 05:30 |
| 21 | AD | 5 | 5 | F | 95 | 14:30 |
| 22 | AD | 5 | 5 | F | 85 | 16:00 |
| 23 | AD | 6 | 5 | F | 88 | 13:30 |
| 24 | AD | 5 | 4 | F | 83 | 10:45 |
| 25 | AD | 5 | 5 | F | 85 | 05:00 |

**Table 1. Human postmortem hippocampal samples.** PMD, postmortem delay; M, male; F, female.

| **Antibody** | **Host** | **Dilution** | **Source** |
| --- | --- | --- | --- |
| APP | Rabbit | 1:1000 | Novus Biologicals, #NBP2-62566 |
| GFP | Chicken | 1:1000 | Synaptic systems, #132006 |
| GFAP | Mouse | 1:1000 | Sigma, #G3893 |
| GFAP | Rabbit | 1:500 | Dako, #GA52461 |
| Iba1 | Mouse | 1:500 | Wako, #09-19741 |
| RTP801/REDD1 | Rabbit | 1:100 | Proteintech, #10638-1-AP |
| AlexaFluor488 anti-chicken IgY | Goat | 1:500 | Thermo Fisher Scientific, #A11039 |
| AlexaFluor555 anti-mouse IgG | Goat | 1:200 | Thermo Fisher Scientific, #A21424 |
| AlexaFluor555 anti-rabbit IgG | Goat | 1:200 | Thermo Fisher Scientific, #A21429 |
| AlexaFluor647 anti-mouse IgG | Goat | 1:200 | Thermo Fisher Scientific, #A21236 |
| AlexaFluor647 anti-rabbit IgG | Goat | 1:200 | Thermo Fisher Scientific, #A21245 |
| AlexaFluor555 anti-rabbit IgG | Donkey | 1:600 | Thermo Fisher Scientific, #A32794 |

**Supplementary table 2.** Antibodies used for immunofluorescence.

| **Antibody** | **Host** | **Dilution** | **Source** |
| --- | --- | --- | --- |
| GFAP | Rabbit | 1:1000 | Dako, #GA52461 |
| GFP | Rabbit | 1:1000 | Thermo Fisher Scientific, #A-11122 |
| RTP801/REDD1 | Rabbit | 1:500 | Proteintech, #10638-1-AP |
| Iba1 | Mouse | 1:1000 | Wako, #019-19741 |
| Synaptophysin | Mouse | 1:1000 | Synaptic Systems, #101011 |
| TrkB | Mouse | 1:1000 | BD Biosciences, #610102 |
| SV2a | Mouse | 1:1000 | Santa Cruz Biotechnology, #sc-376234 |
| Total Akt | Rabbit | 1:1000 | Cell Signaling Technologies, #4691 |
| P-Akt Ser473 | Rabbit | 1:1000 | Cell Signaling Technologies, #4691 |
| P-S6 Ser235/236 | Rabbit | 1:1000 | Cell Signaling Technologies, #4858 |
| Total S6 | Mouse | 1:1000 | Cell Signaling Technologies, #2317 |
| P-mTOR Ser2448 | Rabbit | 1:1000 | Cell Signaling Technologies, #2971 |
| Total mTOR | Rabbit | 1:1000 | Cell Signaling Technologies, #2972 |
| NLRP1 | Rabbit | 1:1000 | Novus, #NBP1-54899 |
| NLRP3 | Rabbit | 1:1000 | Cell Signaling Technologies, Mouse inflammasome kit #20836T |
| Procaspase1 | Rabbit | 1:500 |  |
| Cleaved caspase 1 | Rabbit | 1:500 |  |
| ASC-TM1 | Rabbit | 1:500 |  |
| AIM2 | Rabbit | 1:500 |  |
| Cleaved IL-1β, | Rabbit | 1:500 |  |
| HRP-β-actin | Mouse | 1:100.000 | Sigma, #A3854 |
| Anti-mouse IgG | Goat | 1:10.000 | Thermo Fisher Scientific, #31430 |
| Anti-rabbit IgG | Goat | 1:10.000 | Thermo Fisher Scientific, #31460 |

**Supplementary table 3.** Antibodies used for western blotting.
