# Supplementary figures and images for: RTP801/REDD1 contributes to neuroinflammation severity and memory impairments in Alzheimer’s disease
Source: Cell Death Dis. 2021 Jun 15;12(6):616. doi: 10.1038/s41419-021-03899-y (PMC8206344; doi:10.1038/s41419-021-03899-y)

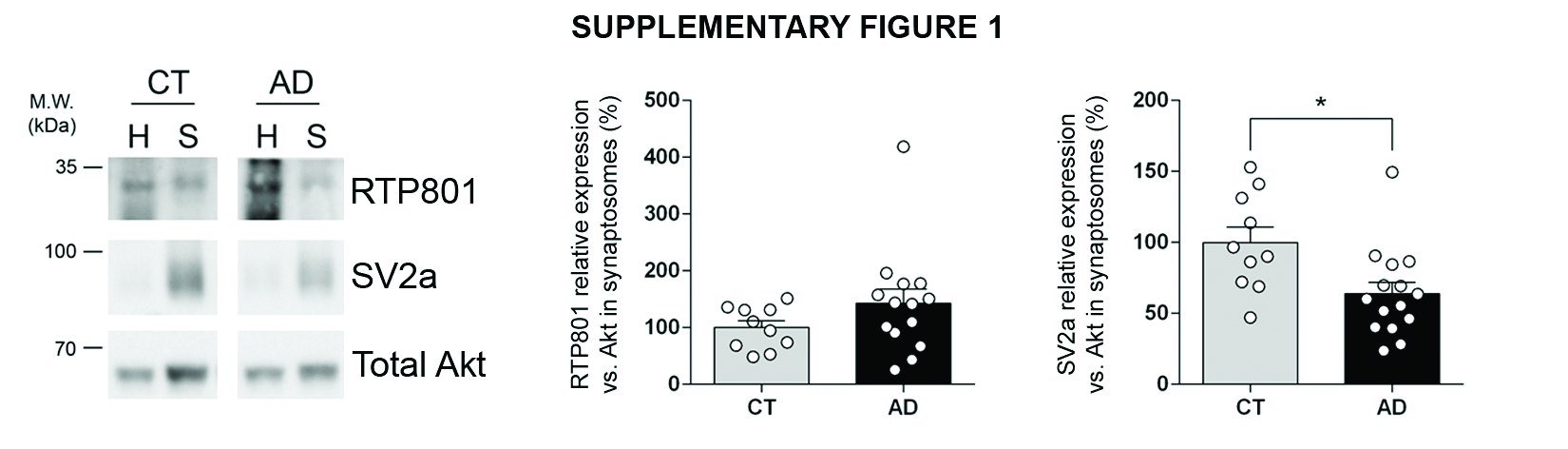

Supplement: Supplementary file 3 — Supplementary Figure 1 [file 41419_2021_3899_MOESM3_ESM.tif]

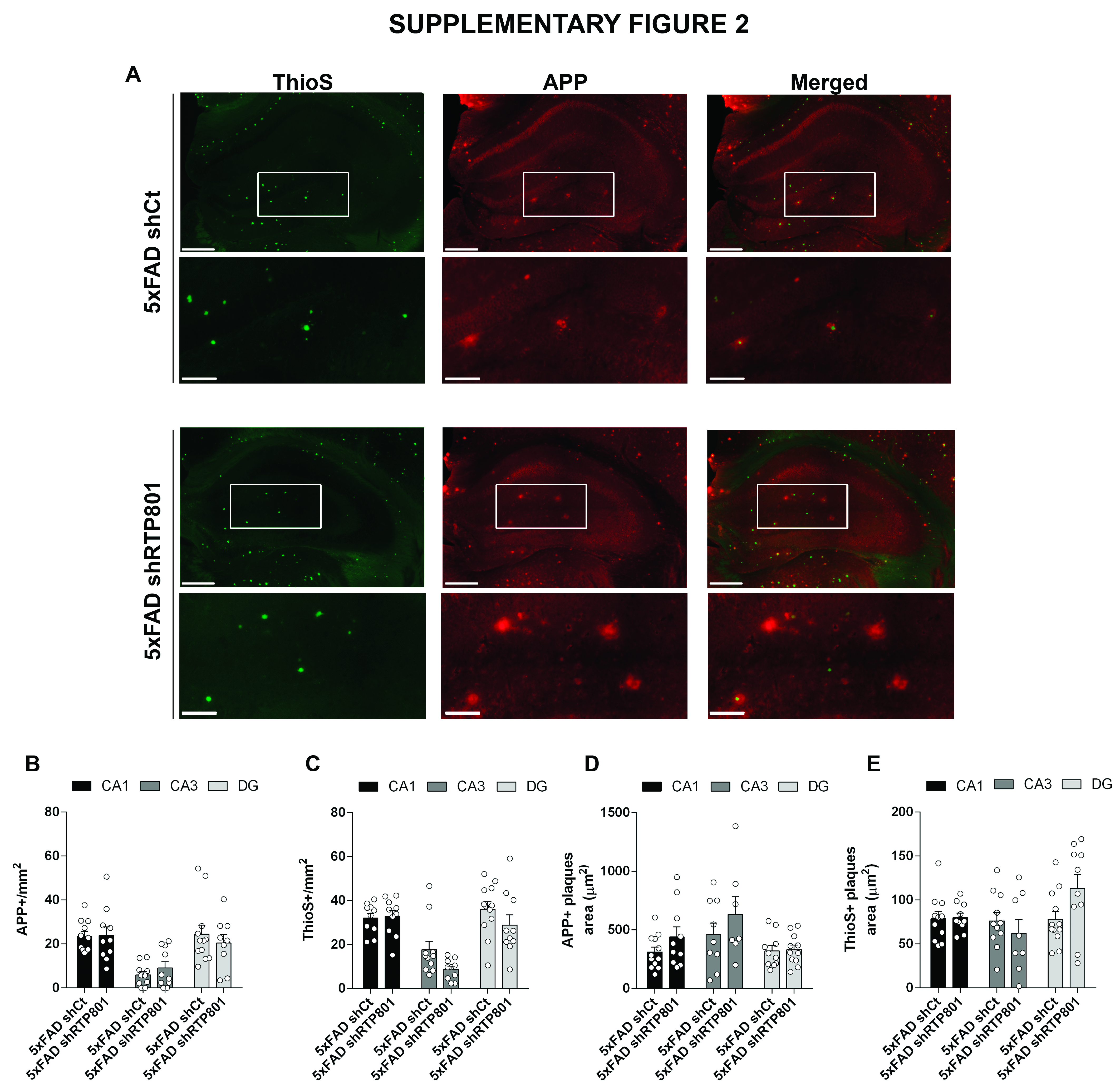

Supplement: Supplementary file 4 — Supplementary Figure 2 [file 41419_2021_3899_MOESM4_ESM.tif]

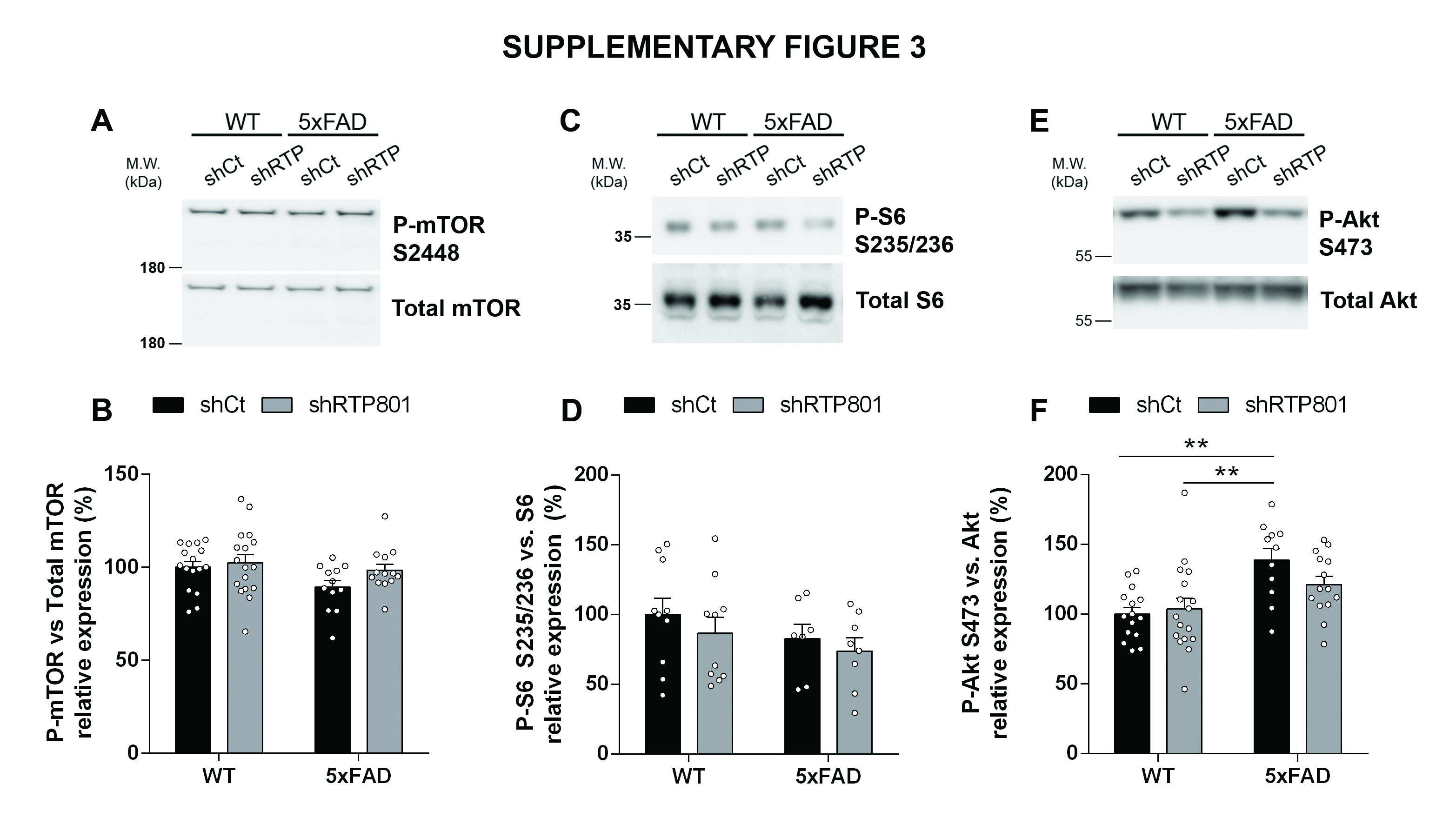

Supplement: Supplementary file 5 — Supplementary Figure 3 [file 41419_2021_3899_MOESM5_ESM.tif]

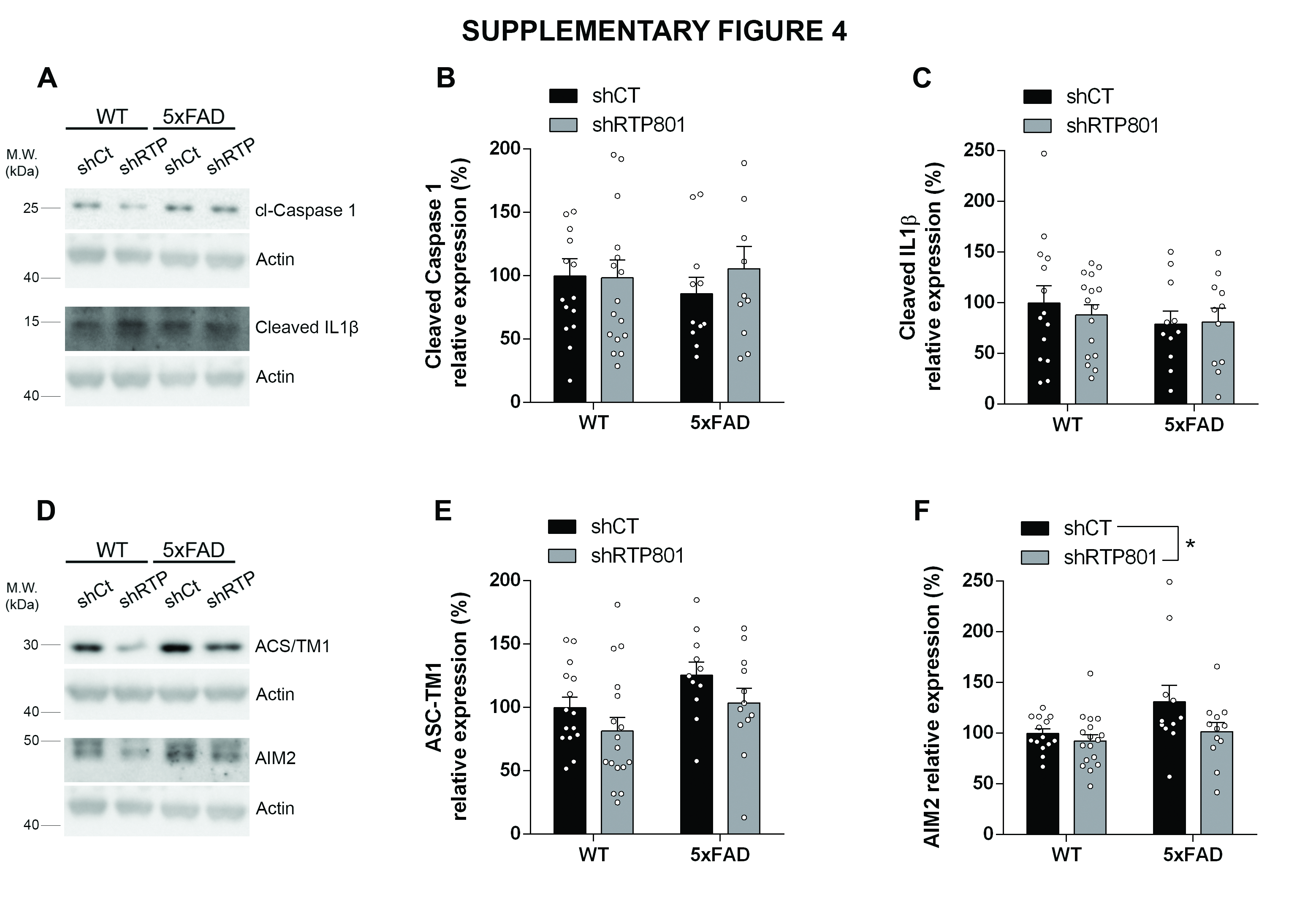

Supplement: Supplementary file 6 — Supplementary Figure 4 [file 41419_2021_3899_MOESM6_ESM.tif]
